# Effect of Single and Dual Modifications on Properties of Lotus Rhizome Starch Modified by Microwave and *γ*-Irradiation: A Comparative Study

**DOI:** 10.3390/foods11192969

**Published:** 2022-09-22

**Authors:** Ankita Chandak, Sanju Bala Dhull, Prince Chawla, Melinda Fogarasi, Szabolcs Fogarasi

**Affiliations:** 1 Department of Food Science and Technology, Chaudhary Devi Lal University, Sirsa 125055, India; 2 Department of Food Technology and Nutrition, Lovely Professional University, Phagwara 144411, India; 3 Department of Food Engineering, University of Agricultural Sciences and Veterinary Medicine of ClujNapoca, CaleaMănăstur 3–5, 400372 Cluj-Napoca, Romania; 4 Department of Chemical Engineering, Faculty of Chemistry and Chemical Engineering, Babeş-Bolyai University, 11 Arany Janos Street, 400028 Cluj-Napoca, Romania; 5 Interdisciplinary Research Institute on Bio-Nano-Sciences, Babeş-Bolyai University, 42 Treboniu LaurianStreet, 400271 Cluj-Napoca, Romania

**Keywords:** lotus rhizome starch, microwave, *γ*-irradiation, dual modification, modification sequence

## Abstract

A comparative study between two novel starch modification technologies, i.e., microwave (MI) and *γ*-irradiation (IR), is of important significance for their applications. The objective of this work is to compare the changes in lotus rhizome starch (LRS) subjected to single modifications by MI (thermal treatment) and IR (non-thermal treatment), and dual modification by changing the treatment sequence, i.e., microwave followed by irradiation (MI-IR) and irradiation followed by microwave (IR-MI). The amylose content of native and modified LRS varied from 14.68 to 18.94%, the highest and lowest values found for native and MI-LRS, respectively. IR-treated LRS showed the lowest swelling power (4.13 g/g) but highest solubility (86.9%) among native and modified LRS. An increase in light transmittance value suggested a lower retrogradation rate for dual-modified starches, making them more suitable for food application at refrigeration and frozen temperatures. Dual-modified LRS showed the development of fissures and dents on the surface of granules as well as the reduction in peak intensities of OH and CH_2_ groups in FTIR spectra. Combined modifications (MI and IR) reduced values of pasting parameters and gelatinization properties compared to native and microwaved LRS and showed improved stability to shear thinning during cooking and thermal processing. The sequence of modification also affected the rheological properties; the G′ and G″ of MI-IR LRS were lower (357.41 Pa and 50.16 Pa, respectively) than the IR-MI sample (511.96 Pa and 70.09 Pa, respectively), giving it a soft gel texture. Nevertheless, dual modification of LRS by combining MI and IR made more significant changes in starch characteristics than single modifications.

## 1. Introduction

Starch is an edible, biodegradable, renewable, inexpensive, and abundantly available natural polymer [1,2]. It has a wide range of industrial uses, including in food, pharmaceuticals, paper, packaging, and textiles [3]. It is used as a main constituent in many starch-rich foods such as pasta, noodles, and bread [4]. Industrial starches are most commonly isolated from wheat, rice, maize, potato, cassava, etc., but the demand for novel starch sources has increased recently [5]. Lotus (*Nelumbo nucifera* Gaertn.) is an aquatic perennial of the *Nelumbonaceae* family, mainly grown in South Korea, China, India, Thailand, Australia, and Japan [1]. Lotus rhizome contains around 10–20% starch on a fresh weight basis with no distinctive flavor or odor. Lotus rhizome starch (LRS) can be used as an additive in preparing different foods such as breakfast, confectionary, and fast foods, especially suitable for children and seniors [6]. Compared to most commercial starch sources such as cereals and tubers, lotus rhizome grows in a completely different environment. This can affect the properties and functionalities of LRS, as described by several subsequent studies on its isolation and characterization [7,8]. However, limited studies have been carried out on the characteristic changes in LRS by modification, which can be due to the fact that lotus rhizome is a relatively exotic source of starch as compared to wheat, corn, tapioca, and potato [8,9,10].

Native or unmodified starches do not have the desired characteristics for industrial use due to some of their inherent limitations, such as weak heat and shear resistance, easy thermal decomposition, higher viscosity, easy retrogradation, and low light transmittance [4,11]. These shortcomings of native starch can be improvised using a single or combination of different modification methods, including chemical, enzymatic, and physical treatments, making it desirable for various industrial applications [12]. Due to chemical residue in chemical processes and the high cost of enzymatic treatments, physical methods, which are generally recognized as safe, are preferred, especially in food applications [13]. Additionally, the emerging concept of “green technology” for environmentally friendly applications favored the use of physical methods for starch modifications [14]. Among these, microwave and irradiation treatments have been used nowadays due to their simplicity, higher efficiency, no chemical use, and environmentally friendly nature [1,14,15].

Unlike conventional thermal processes, microwave heating is a multi-physical field phenomenon that involves the non-ionizing radiations generated by a high-frequency magnetic field. These radiations cause dipole oscillation and friction in the substance, resulting in direct heat conversion, known as the dielectric effect. The process has the advantages of faster heating and processing, high penetration, and energy efficiency [16]. Microwave irradiation appears to be capable of causing local melting of crystalline starch, hence facilitating recrystallization into diverse crystal forms [17]. As a result, microwave technology has the potential to be a valuable tool for modifying the retrogradation behavior and crystal structure of starch in a regulated manner.

On the other hand, *γ*-irradiation treatment involves direct exposure to electromagnetic rays. It does not cause a significant temperature increase, involves little sample preparation, is faster, and does not rely on any form of catalyst [18]. The primary mechanism of *γ*-irradiation processing has been shown to produce free radicals that can induce structural changes and starch fragmentation resulting in physicochemical changes in starchy food, such as low viscosity and high water solubility [18].

In some instances, a single modification of many starches has been found insufficient to optimize their functionality that could meet various industrial needs [11,19]. Therefore, dual modifications have been introduced to create starches with novel functionality to expand applications in different areas. Several researchers evaluated the effects of the modification of starch using microwave and irradiation individually, but limited studies focused on the comparative evaluation of the impact of the single and dual modifications. Additionally, the effect of the change in the modification sequence on dual-modified starches is less reported. In this study, the effect of microwave (thermal) and *γ*-irradiation (non-thermal) treatments on LRS when applied in combination was compared with individual treatments. Additionally, dual modifications were carried out by changing the sequence of thermal and non-thermal treatment to observe their effect on different characteristics of starch.

## 2. Materials and Methods

### 2.1. Materials

Lotus rhizomes were procured from the local market in Sirsa, Haryana, India. All the chemical reagents used were of analytical grade.

#### 2.1.1. Starch Isolation

The starch was isolated from lotus rhizomes following the method described by Singh et al. [20] after little modification. Briefly, mature lotus rhizomes harvested in the later swelling stage were peeled, cut, and pulverized with ice-cold water using a blender (Sujata Powermatic Plus, New Delhi, India). The resulting slurry was screened through cheesecloth. In a blender (Sujata Powermatic Plus, New Delhi, India), the fibrous residue was further homogenized and squeezed to release the starch trapped inside. The starch slurry was filtered through sieves (0.250, 0.150, 0.100, 0.075, and 0.045 mm) and left to settle for 4–5 h. Then, distilled water was added to the settled starch layer, mixed, and centrifuged (Eltek, TC 8100F, Mumbai, MH, India) at 3200× *g* for 10 min and the supernatant was withdrawn. The sediment was washed five times to purify lotus rhizome starch (LRS), dried in a hot-air oven (NSW-143, New Delhi, India) at 45 °C for 24 h, pulverized, and sieved through 100-mesh size, and the fine powder was stored for further analysis.

#### 2.1.2. Proximate Composition

The proximate composition (moisture, fat, protein, crude fiber, and ash content) of the native LRS was analyzed using AOAC methods [21]. The samples were analyzed in triplicates.

### 2.2. Single and Dual Modification of Lotus Rhizome Starch (LRS)

#### 2.2.1. Modification by Microwave Treatment (MI)

The method of Deka and Sit [22] was followed to modify the lotus rhizome starch by microwave (MI) treatment. Water was sprayed onto powdered LRS (50 g) to adjust its moisture content to 30% and analyzed using a moisture analyzer (HB43-S, Mettler Toledo, New York, NY, USA). The starch sample was kept overnight under refrigerated (REMI, RLR-300, Mumbai, MH, India) conditions for uniform moisture distribution. The moisture-calibrated starch samples were then subjected to MI processing at 2450 MHz and 180 W for 3 min using a microwave (Model MH7046, LG, Kuala Lumpur, Malaysia). The microwaved LRS was then dried, powdered, sieved, and stored for further studies.

#### 2.2.2. Modification by *γ*-Irradiation (IR)

The LRS samples (50 g) were treated with *γ*-irradiation using the method of Punia et al. [1]. At room temperature (23 ± 2 °C), the packed LRS was exposed to a single dose of 10 kGy gamma irradiation utilizing cobalt-60 as the source irradiator (Gamma Chamber 5000, BRIT, Government of India, ANSI-N433.1, India).

#### 2.2.3. Dual Modification by Microwave Followed by *γ*-Irradiation (MI-IR)

The dried microwaved LRS (MI) (50 g) was subjected to *γ*-irradiation treatment as described earlier. After irradiation, a dual-modified LRS sample (MI-IR) was powdered, sieved, and stored until further studies.

#### 2.2.4. Dual Modification by *γ*-Irradiation Followed by Microwave (IR-MI)

The *γ*-irradiated LRS (IR) (50 g) was subjected to microwave treatment as described earlier. After microwaving, a dual-modified LRS sample (IR-MI) was dried, powdered, and stored for further studies.

### 2.3. Amylose Content

The amylose content of native and modified LRS was determined using the method described by Williams et al. [23] with minor modification. The absorbance of the solution was noted at 625 nm using a spectrophotometer (Systronics-2203, Ahmadabad, India). A standard curve made from amylose and amylopectin blends was used to determine the amylose content in triplicate.

### 2.4. Swelling Power and Solubility

The swelling power (SP) and solubility of native and modified LRS were determined using the method published by Leach et al. [24] with minor modifications. Starch (1 g) was mixed with distilled water (99 mL), heated for 1 h at 90 °C, cooled in an ice-water bath for 1 min, and equilibrated (25 °C, 5 min), then centrifuged (Eltek, TC 8100F, Mumbai, MH, India) at 3000× *g* for 30 min. The supernatant was then poured into a pre-weighed moisture dish, dried in a hot-air oven (NSW-143, New Delhi, India) at 100 °C, cooled, and weighed again. Additionally, the weight of swollen starch sediment was noted. The swelling power (g/g on a dwb) and solubility were determined as follows:

Solubility (%) = 100× (Wt. of dried supernatant)/Wt. of dry LRS

SP (g/g) = Wt. of sediment (g)/Wt. of dry LRS (g)

### 2.5. Transmittance (%)

The transmittance of native and modified LRS was measured using the method of Perera and Hoover [25]. Briefly, LRS aqueous suspension (1%) was heated (90 °C, 1 h) in a water bath (Brookfield, TC-202, Harlow, UK) with continuous stirring, then cooled (30 °C, 1 h) and kept refrigerated (REMI, RLR-300, Mumbai, MH, India) at 4 °C for five days. After every 24 h, the absorbance was measured against a water blank at 640 nm to calculate the transmittance.

### 2.6. Morphological Properties

Scanning electron micrography (scanning electron microscope, JSM-6100, Jeol, Peabody, MA, USA) was carried out with a drop of starch–ethanol suspension (1%) applied to an aluminum stub using both-sided adhesive tape, at an accelerating potential of 10 kV with a magnification power 1000×.

### 2.7. FTIR Analysis

Attenuated total reflection–Fourier-transform infrared spectroscopy (ATR-FTIR, Bruker ALPHA 200229, Bremen, Germany) with diamond crystal cell ATR and inbuilt IR -Solution software at 2 cm^−1^ resolution was used to obtain the vibrational spectra of all LRS samples. With the air taken as a background, 45 scans of each sample were carried out in the mid-infrared band 4000–450 cm^−1^. Each sample was repeated at least three times to obtain spectra in terms of absorption.

### 2.8. X-ray Diffraction

Using an X-ray diffractometer (Rigaku Miniflex, Rigaku Co., Tokyo, Japan) operating at 45 kV and 40 mA, a wavelength of 0.154 nm was used to record the X-ray pattern of LRS samples. Diffractograms were acquired at 25 °C over a 2θ range of 10–50 with a step size of 0.02 and a sampling interval of 10 s. The relative crystallinity of the LRS was calculated using the equation:

Crystallinity (%) = (Area of diffraction peak)/(Total area) × 100

### 2.9. Pasting Properties

A Rheometer (MCR-52, Anton Paar, Graz-8054, Austria) with an in-built starch cell was used to examine the pasting properties of native and modified LRS samples. The starch suspension (1.2 g LRS + 13.8 g distilled water) was equilibrated at 50 °C for 1 min, then heated from 50 to 95 °C at 6 °C/min, and then held at 95 °C (2.7 min). The suspension was then cooled to 50 °C at the same rate and held for 2 min (at 50 °C). Pasting temperature (PT), pasting viscosities (peak viscosity (PV), breakdown viscosity (BV), trough viscosity (TV), setback viscosity (SV), and final viscosity (FV)) were obtained from the pasting graph.

### 2.10. Thermal Properties

The thermal properties of LRS samples were determined using a Differential scanning calorimeter (DSC) (Discovery-25, TA Instruments, New Castle, DE, USA). A starch–water suspension having 70% water was loaded into a 40 µL aluminum pan, hermetically sealed, and equilibrated for 1 h at room temperature. The sample pan was then heated from 20 to 250 °C at a rate of 10 °C/min. The onset temperature (*T*_o_), peak temperature (*T*_p_), conclusion temperature (*T*_c_), and gelatinization enthalpy (∆H_gel_) were all determined automatically.

### 2.11. Dynamic Rheological Behavior

The frequency sweep tests of all LRS samples were performed as per the method of Kaur and Singh [26] with slight modifications. Starch slurry (15% *w*/*w* in distilled water) was prepared, stirred manually, heated (90 °C, 30 min), and stirred again for 3 min. After cooling at room temperature and loading on the rheometer ram, the frequency sweep measurements were performed from 0.1 to 100 rad/s at 25 °C and 2% strain (as found from the linear viscoelastic range 0.1–50% for all starches) using the Modular Compact Rheometer (Anton Paar, Austria) equipped with a parallel plate system (4 cm diameter). The G′ (storage modulus), G″ (loss modulus), and tan δ (loss tangent) were recorded at an angular frequency of 6.28 rad/s.

### 2.12. Statistical Analysis

The data presented in the tables were analyzed in triplicate, and single-factor analysis of variance (ANOVA) was carried out for the experimental results. Duncan’s multiple range tests (DMRT) using SPSS 20.0 software (IBM Analytics, IBM Corporation, Armonk, NY, USA) were used to determine the significant differences between the data.

## 3. Results

### 3.1. Proximate Composition of Native LRS

The total yield of native LRS was 4.0 g/100 g and the proximate composition showed a moisture content of 10.32 ± 0.58% (wet basis), while fat, protein, crude fiber, ash content, and purity were 0.20 ± 0.03%, 0.66% ± 0.11%, 0.27 ± 0.05%, 0.19 ± 0.01%, and 98.25 ± 1.15% (dry basis), respectively.

### 3.2. Amylose Content of Native and Modified LRS

The amylose content is a primary quality characteristic of starches which governs their different properties and ultimate end-use purposes. The amylose content of native, single-modified (MI, IR), and dual-modified (MI-IR and IR-MI) LRS was found to be 18.94%, 14.68%, 16.55%, 17.58%, and 18.86%, respectively (Table 1). The amylose content of processed starches reduced after all treatments in comparison to the native sample, but the decrease was higher for single-modified starches. Additionally, microwaves showed a higher decrease than *γ*-irradiation, which could be due to the partial degradation/depolymerization of amylose resulting from heat generated during microwaving. Furthermore, increased amylose leaching in microwaved samples favored its complexing with lipids and proteins [14,27], ultimately decreasing the total amylose content of the MI-treated starches. On the other hand, ionizing radiations such as *γ*-irradiation can cause random scission in the glucosidic chains and produce short amylose units and linear chains from the branched amylopectin [18]. These short-chain units have a low ability to complex with iodine, thereby showing lower values for amylose content. A decrease in amylose content was also reported for lotus seed starch [11], brown rice starch [28], and arrowhead tuber starch [29] after irradiation. Additionally, it seemed that the MI-IR modification reduced the amylose content more than the IR-MI modification. When microwaving was carried out before irradiation, the amylose degradation might be more due to high heat generation during microwave heating, whereas in IR-MI treatment, irradiation facilitated the release of linear short chains and amylose which compensated for the excess amylose degradation during MI treatment.

### 3.3. Swelling Power and Solubility

Swelling describes the capability of starch granules to trap and hold water within their structure and mainly depends on the amylopectin content of starches than their amylose content [30]. The results for swelling power (SP) of native, single-, and dual-modified LRS are shown in Table 1. The SP and solubility of starch are indicators of the degree of interaction between starch chains within the amorphous and crystalline domains [11]. The native LRS showed the highest SP (23.82 g/g), which decreased to 19.50, 4.13, 10.00, and 9.71 g/g after MI, IR, MI-IR, and IR-MI treatments, respectively. Oyeyinka et al. [31] and Punia et al. [1] also reported reduced SP after the microwave heating of Bambara groundnut starch and the *γ*-irradiation of lotus seed starch, respectively. The reduction in SP after modification involving heat treatments (microwave) can be due to the destruction of the starch granular structure, resulting in amylopectin chain degradation. The heating presumably also results in re-arrangements leading to the more random distribution of crystalline regions within the starch granules [32]. Furthermore, starch gelatinization and protein denaturation at high temperatures also cause hindrance to the diffusion of water in the starch matrix. Therefore, microwaved LRS can be suitable for food products requiring lesser swelling such as noodles.

In comparison to MI treatment, IR resulted in more reduction in the SP of modified starches. This reduction in swelling after IR treatment might be due to the amylopectin fragmentation by free radicals and reduction in starch and denatured protein matrix, preventing water diffusion into the starch matrix [33,34]. Kerf et al. [35] also observed a significant decrease in the amylopectin fraction of different starches upon irradiation. The higher decrease in SP in irradiated starch (both single as well as dual) also explains the production of short chains amylose due to random scissions in amylopectin branched structure. However, the amylose content analyzed by iodine-binding spectrophotometry does not show increased values for irradiated LRS samples which could be due to their low iodine-binding efficiency (as discussed in Section 3.2). As the decrease in SP could prevent the bursting of starch granules, it can be beneficial to improve the textural quality of foods upon cooking. The leaching of amylose and amylopectin from starch granules, followed by maximum swelling, determines starch solubility. The solubility of native, as well as single- and dual-modified samples (MI, IR, MI-IR, and IR-MI), increased significantly (*p ≤* 0.05) with increasing temperature from 60 to 90 °C (data not shown). At 90 °C, the highest and the lowest solubility were recorded for single-modified IR-starch (86.9%) and native LRS (9.63%), respectively. During MI treatment, starch granules rupture and are exposed due to partial gelatinization which helps in the leaching and solubilizing of amylose and low-molecular-weight amylopectin resulting in the increased solubility of modified starches [36].

Additionally, a manifold increase in solubility was observed in dual-modified starches involving *γ*-irradiation. The increase in solubility by IR treatment could be related to the degradation of starch structures and the production of low-molecular-weight fractions having a greater affinity for water, thereby elevating the solubility [15]. Mohd Adzahan et al. [37] also reported increased amylose leaching for sago and tapioca starches with increasing exposure to radiations. Previous studies reported comparative results after *γ*-irradiation treatment of different starches [1,15]. Some previous studies also reported an increase in solubility after *γ*-irradiation treatment of lotus seed starch and talipot starch.

### 3.4. Water Absorption Capacity (WAC) and Oil Absorption Capacity (OAC)

The ability of solids (starch, protein, and flour) to entrap water or oil against gravity define their water absorption capacity (WAC) and oil absorption capacity (OAC). The WAC of native and modified LRS ranged from 1.62 to 2.00 g/g (Table 1). It was significantly (*p ≤* 0.05) increased after single and dual modifications; the highest value was observed for IR-MI samples. Microwave and *γ*-irradiation caused starch degradation to smaller/simple sugars such as dextrin, maltose, glucose, etc., which have a higher affinity for water than starch [7,38]. High amylose content also adversely affects the WAC of starches [7]. The modified LRS samples showed higher OAC than native (1.30 g/g), the highest value (1.89 g/g) exhibited by MI-IR LRS. An increase in OAC might be due to the destruction of the starch structure upon MI and IR treatments, resulting in the exposure of more hydrophobic portions of the amylose–lipid complex [39]. Similarly, MI-treated lotus seed starch exhibited higher OAC than native starch [30]. Single- and dual-modified starches have altered functional characteristics (swelling, solubility, WAC, and OAC), and therefore can help prepare viscous foods such as jellies, soups, custard, and puddings, and some bakery products such as cakes and cookies.

### 3.5. Transmittance (%)

**Figure 1:**
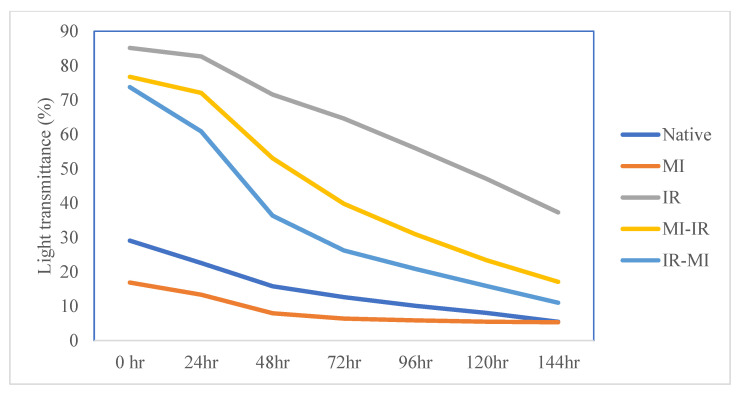
Effect of storage duration on light transmittance (%) of native and modified lotus rhizome starch pastes.

The change in transmittance of all LRS samples is shown in Figure 1. The part of incidence light that passes through the starch at a specific wavelength is known as light transmittance. It gives information on how starch paste behaves when light travels through it. A high transmittance value shows high paste clarity which is a desirable characteristic of starch. Light transmittance showed a general decreasing trend with storage time for native, single-modified (MI and IR), and dual-modified (MI-IR and IR-MI) LRS samples and the value ranged from 29.07 to 5.47%, 16.88 to 5.32%, 85.16 to 37.30%, 76.75 to 17.09%, and 73.74 to 11.01%, respectively. The reduction in values of light transmittance during storage might be due to starch retrogradation, which involves re-associating broken bonds in an ordered structure. Several previous studies also showed comparative results for native and modified (microwaved or irradiated) starches [1,15,40].

Upon microwaving, the light transmittance decreased significantly (*p* ≤ 0.05) compared to the native counterpart. The degradation of starch molecules in different-molecular-weight amylose may contribute to the packing of the starch paste after MI treatment, increasing the opacity and affecting the paste clarity [41]. Additionally, gelatinization during MI processing might ease the amylose release, which caused MI-modified LRS to have lower paste clarity. Zailani et al. [42] also observed decreased paste clarity after the microwave processing of washed and cold-soaked sago starch. Low paste clarity of starch affects consumers’ preference by affecting the appearance of foods (e.g., puddings and drinks) and producing vague products.

*γ*-irradiation improved the light transmittance of LRS after single and dual modification, with the single IR-treated sample showing the highest values (Figure 1). It was suggested that the increase in paste clarity of IR-treated starch could be owed to the generation of carboxylic groups after IR-treatment resulting in increased water-holding capacity [43]. Comparable results were also reported in IR-treated lotus seed starch and talipot palm starch [1,15]. The light transmittance of the dual-modified starches, MI-IR and IR-MI, was significantly (*p* ≤ 0.05) higher than that of the native and MI-treated LRS. The synergistic action of MI and IR might decrease swollen starch granules, followed by the gelling and splitting of amylopectin chains, thus increasing light transmittance. Furthermore, due to reduced retrogradation, the light transmittance of dual-modified LRS was enhanced. The enhanced paste clarity suggests that modified starch could be used in different frozen food products and pie fillings.

### 3.6. Granular Morphology

**Figure 2:**
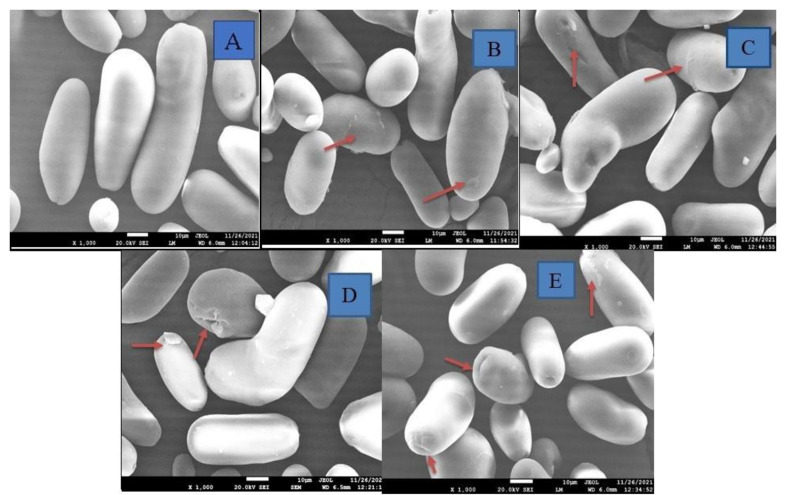
Morphological properties of native and modified lotus rhizome starches. (**A**)—Native; (**B**)—Microwaved (MI); (**C**)—Irradiated (IR); (**D**)—Microwaved–Irradiated (MI-IR); (**E**)—Irradiated–Microwaved (IR-MI).

A scanning electron microscope (SEM) plays a vital role in examining and comparing the surface characteristics of native and modified starch granules. The SEM micrographs of native, single-modified (MI and IR), and dual-modified (MI-IR and IR-MI) LRS are presented in Figure 2A–E. Native LRS granules had a smooth surface, and varied in shape from round and oval to elliptical, and size from small to large. Small starch granules had a round shape, whereas larger granules were oval to elliptical. However, minor alterations were noticed in single-modified MI and IR, which included increased surface roughness, fissures, and dents on starch granules. The surface of the MI samples became rough, which can be attributed to the rapid pressure built up within starch granules during microwave heating, resulting in their rapid expansion. However, this rapid expansion of granules could not be sustained by the hydration of the granules; therefore, MI-processed starch granules collapsed or sometimes ruptured instead of swelling [44]. In IR samples, fissures and dents were observed, which can be due to the disintegration of starch granules by high-energy *γ*-irradiation. Similar morphological effects after *γ*-irradiation were reported on talipot stem starch [15] and lotus seed starch [1]. Some factors such as irradiation dose, starch source, crystalline pattern (B-type starches are more resistant to *γ*-irradiation than C-type), and moisture content might affect the resistance of starch granules against rupturing [45]. The surface morphology of starch granules changed more drastically after dual modifications (MI-IR and IR-MI) compared to single modification and showed a rougher surface with more dents, fissures, and even rupturing of some granules. In addition, MI-IR LRS showed more surface and granule damage than the IR-MI sample. MI-processing before IR-processing resulted in a weak and hardly porous starch granule (due to rapid inside-pressure development) which cannot withstand the high penetration power of *γ*-irradiation, resulting in its rupture.

### 3.7. FTIR Spectral Analysis

**Figure 3:**
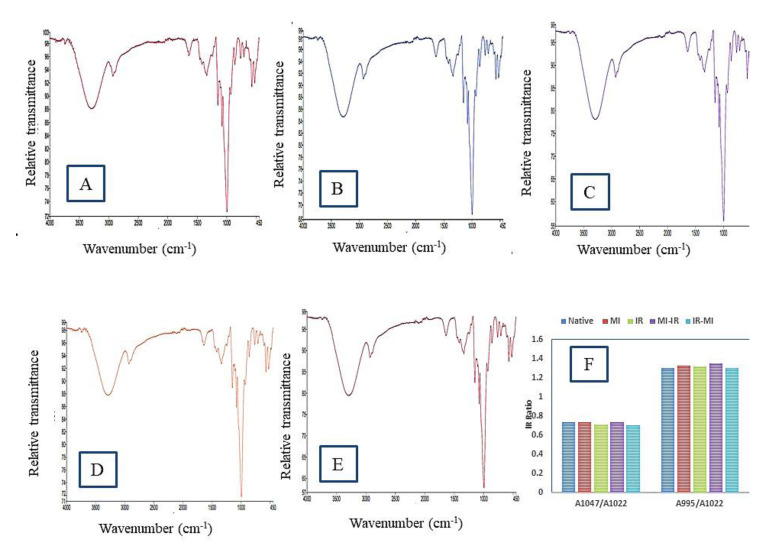
FTIR spectra of native and modified lotus rhizome starches. (**A**)—Native; (**B**)—MI; (**C**)—IR; (**D**)—MI-IR; (**E**)—IR-MI; (**F**)—IR—ratios for absorbance 1047/1022 and 995/1022 of native and modified LRS.

An FTIR spectrum represents the bands corresponding to stretching, bending, and deformation related to the main functional groups present in the starch granule. The FTIR spectra of native and single- and dual-modified LRS are presented in Figure 3A–E. The key bands can be sequentially interpreted and characterized by four main regions of the FTIR spectra involving the wavenumber ranges (i) less than 800 cm^−1^, (ii) 800−1300 cm^−1^ (the fingerprint zone), (iii) 2800–3000 cm^−1^ (C−H stretch zone), and (iv) 3000–3600 cm^−1^ (O−H stretch zone). The main absorption peaks of native LRS were observed around 3292.90, 2932.64, 1644.83, 1337.23, 1076.75, 994.97, and 524.24 cm^−1^. The broad and strong characteristic absorption band around 3292.90 cm^−1^ (native), 3290.81 cm^−1^ (MI), 3293.46 cm^−1^ (IR), 3276.05 cm^−1^ (MI-IR), and 3292.48 cm^−1^ (IR-MI), attributed to-OH stretching [46], showed more exposure to the hydroxyl group due to radiation treatment. The peak at 2932 cm^−1^ by a stretching vibration in all LRS samples showed the presence of CH_2_ deformation [47]. In dual-modified LRS, reduced peak intensity was observed for the peaks corresponding to -OH and CH_2_ groups’ stretching vibration. The intensity of the peak at 1644.83 cm^−1^, corresponding to the H–O–H bending vibration in water molecules of the starch amorphous region [48], was also reduced after dual modification. The peaks at 1337 cm^−1^ and 1149 cm^−1^ were due to CH_2_ bending and C-O-O twisting [49] and CO, C-C stretching [50], respectively. The deep characteristic band at 994 cm^−1^ represented the presence of α-1,4glycosidic linkage and C-O-C stretch [51]. As shown in Figure 3B,C, the patterns of the spectra were not changed and no emergence or loss of characteristic absorption peak was observed after MI and IR treatment, which suggested that microwave and irradiation treatments only exhibited physical effects on the starch samples [52]. However, peak broadening and a reduction in peak intensity were observed for all single- and dual-modified LRS samples, although the degree of change varied. The broadening of peaks can be due to the larger distribution of bond energies and reduction in polymer ordering [53], while damage to starch conformation such as double-helix structure and crystalline regions can be attributed to changes in peak intensities [54].

After MI treatment, the peak intensity decreased significantly and became wider. It is reported that inter- and intra-molecular hydrogen bonding are enhanced during microwave processing which is also evidenced by the increase in the gelatinization onset temperature reported in thermal measurements [55]. The same trend was also reported in microwave-treated millet starch [56]. *γ*-irradiation treatment did not cause a remarkable change in the intensity of absorption peaks which might be due to the low moisture present in the starch or the lower dose of irradiation used in the processing of LRS [1]. Howbeit, upon dual treatment with microwave and *γ*-irradiation, the destruction of chemical bonds by high energy radiations was signified by the changes in the FTIR spectra of the OH and CH_2_ stretching and H–O–H and C–O–H bending vibration. During dual modification, unstable reactive agents (free H-atoms, aqueous electrons, and hydroxyl free radicals) were produced from the water present in the starch, which decreased the peak intensities corresponding to OH and CH_2_ groups [57].

Moreover, the internal changes in the degree of order (DO) which refers to the RC can be calculated using the ratio of 1047/1022, while the degree of double helix (DD) can be calculated by using the ratio of 995/1022, based on the FTIR spectra of starches [58]. The 1047/1022 ratios for all modified starches were lower compared to the native LRS (0.7362). Meanwhile, a higher ratio was observed for the 995/1022 ratio for single- as well as dual-modified LRS than the native counterpart. A lower 1047/1022 ratio for modified starches indicated a possible lower DO presence [59]. During MI processing, partial gelatinization disrupting the ordered crystalline structure of amylopectin possibly reduced the DO of modified starches [42]. On the other hand, the *γ*-irradiation might destruct the crystalline phase through the molecular depolymerization by free radicals which split the amylopectin units and decrease the total crystallinity [48]. The results are in agreement with the findings of XRD analysis where RC decreased for all modified LRS samples (Section 3.8). Meanwhile, a higher 995/1022 ratio shown by modified LRS may suggest a higher proportion of double-helix amylose [59]. The amylose leaching during MI treatment and the production of linear chains from the branched amylopectin during IR treatment increases the chances of more interactions and hydrogen bonding being formed between amylose molecules.

### 3.8. Crystalline Structure

**Figure 4:**
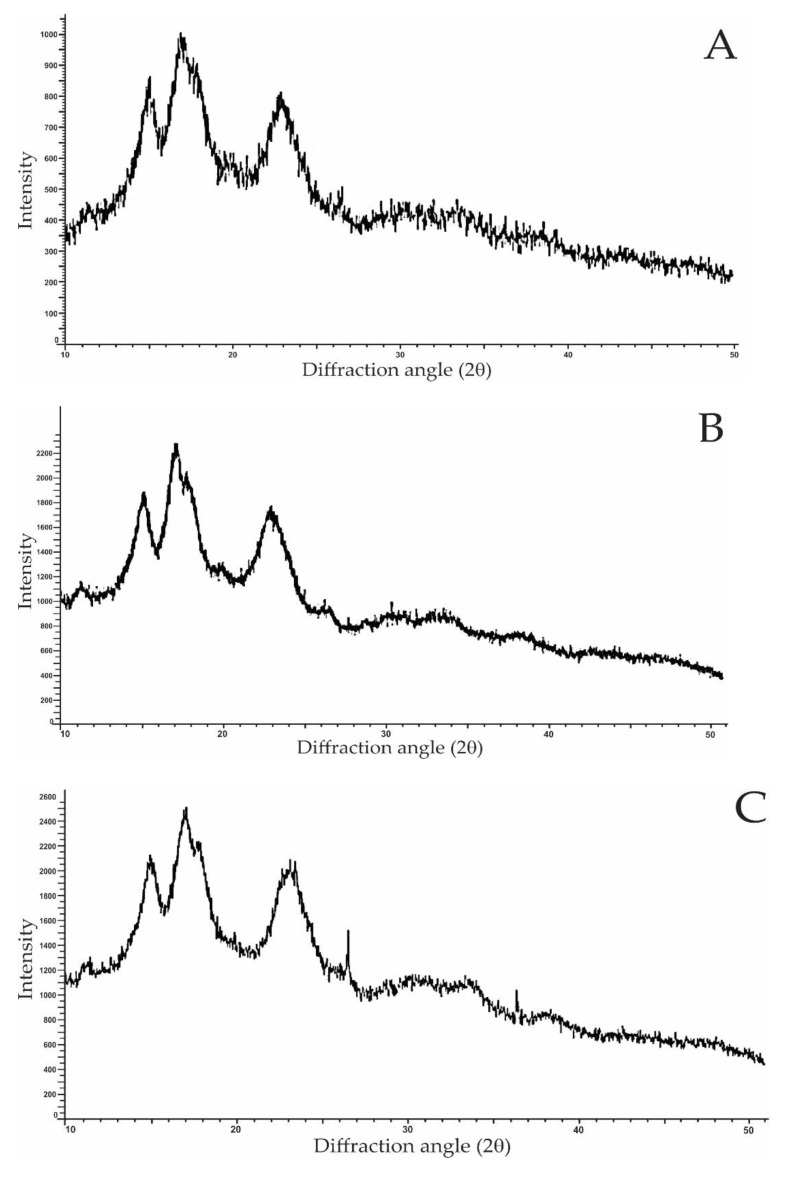

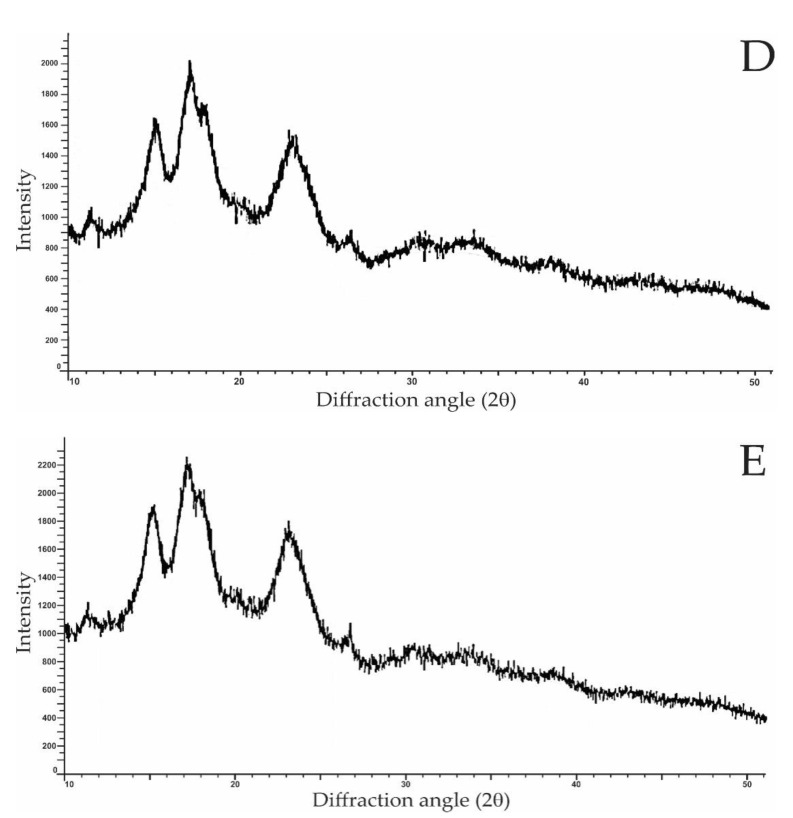
XRD spectra of native and modified lotus rhizome starches (**A**)—Native; (**B**)—MI; (**C**)—IR; (**D**)—MI-IR; (**E**)—IR-MI.

X-ray diffractometry is used to demonstrate the presence and characteristics of the crystalline structure of starch granules. Starch has been demonstrated to have four different crystalline patterns using XRD (A, B, C, or V polymorphs). The packing arrangements of the double helix in amylopectin and the hydration levels are used to distinguish the A and B crystalline patterns. Because the double helix is packed closely, the A-type is less hydrated, whereas the B-type has a more hydrated helical core. Figure 4A–E show the X-ray diffractogram of native and single- and dual-modified LRS. The native and modified LRS did not show significance in their diffraction patterns; however, the peak intensity increased in the XRD patterns of single- and dual-modified samples, suggesting a change in the degree of crystallinity of LRS. The diffractogram of native, single-, and dual-modified LRS showed peaks at an angle of 15°, 23° and a doublet at 17°, 18° (2*θ*), epitomizing a typical type-A starch pattern. Similar results for native LRS (peaks at an angle of 15.07°, 23.09° and doublet at 17.10°, 18.01° (2*θ*)) have been reported earlier [8]. Starch modifications such as microwave and *γ*-irradiation treatment probably degrade the amorphous lamella, promote the debranching of amylopectin, and degrade amylose into smaller molecules. These changes result in reorganization and increase the crystallization of starch molecules, providing XRD patterns with high-intensity peaks. Earlier, Chung and Liu [60] also found no difference in the diffraction pattern of native and irradiation-treated bean and potato starches. Other researchers reported no change in the crystalline pattern of microwave-treated starches [31]. Some factors such as starch moisture level, type (normal or waxy), and modification method used in combination with microwave processing appear to influence these alterations [14].

The relative crystallinity (RC)of native LRS was 28.2%, which was higher than that of single- and dual-modified LRS (Figure 4A–E). The IR-MI LRS showed the lowest relative crystallinity (25.1%), followed by MI-IR (25.8%), IR (26.4%), and MI (27%)-treated samples. Earlier studies using microwaves and other heat treatments found a reduced relative crystallinity of taro starch. It was pointed out that the loss of the crystal region during processing due to hydrogen bond cleavage resulted in the weakening of the peak intensity [22]. A decrease in relative crystallinity has also been reported in *γ*-irradiation-treated lotus seed starch by Punia et al. [1]. This decrease in relative crystallinity may be due to the destruction of long-range ordering linked to the ordered structure of crystalline and amorphous regions and destabilization of the double-helix structure of the amylopectin crystallized region under IR and MI conditions [1,38], and this decrease was more obvious under the dual treatment.

### 3.9. Pasting Properties

**Figure 5:**
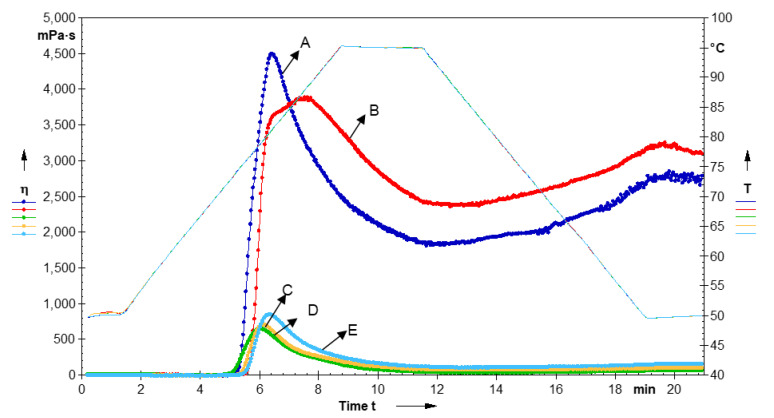
Pasting profile of native and modified lotus rhizome starches. A—Native; B—MI; C—IR; D—MI-IR; E—IR-MI.

The pasting profile of native and modified LRS samples is shown in Table 2 and Figure 5. During heating and gelatinization, the molecular arrangements of starch undergo a radical alteration, with consequent changes in properties. The pasting temperature (PT) is the minimum temperature required to cook the starch. Peak viscosity (PV) represents the highest viscosity of starches at the equilibrium point between swelling and polymer leaching due to an increase in temperature, while breakdown viscosity (BDV) shows the stability of starch granules against increasing temperatures and shear forces. On cooling, the aggregation of amylose molecules resulting in an increased viscosity is indicated by final viscosity (FV), while setback viscosity (SBV) shows the gelation or retrogradation ability of starch [11]. Native LRS showed PT, PV, TV, BDV, FV, and SBV to be 74.3 °C, 4486 mPa.s, 1834 mPa.s, 2652 mPa.s, 2791 mPa.s, and 957 mPa.s, respectively. The MI treatment significantly (*p* ≤ 0.05) decreased PV, BDV, and SBV but a significant (*p* ≤ 0.05) increase was observed in PT, TV, and FV. The reduction in PV after MI treatment can be due to the degradation of polysaccharides and reduction in the swelling power of starch granules [40]. A similar trend for PV was also reported for Indian horse chestnut starch [40]. The lower BDV of MI-treated LRS indicates that swollen granules were more resistant to shear thinning during any mechanical action such as cooking [14,40], while lower SBV indicated its stability toward retrogradation. The *γ*-irradiation significantly (*p* ≤ 0.05) decreased the PT and all pasting viscosities of IR-LRS, which showed the lowest values in comparison to native as well as single (MI) and dual (MI-IR and IR-MI)-modified LRS. The lower PT, PV, and SBV connote decreased resistance to swelling and rupturing of starch granules. A similar decrease has also been reported in lotus seed starch and talipot stem starch [1,15]. This decrease in FV and SBV of IR-treated LRS can be due to a decreased degree of polymerization resulting from the breaking of the starch chain after IR treatment.

The dual-modified (MI-IR and IR-MI) LRS showed significantly decreased (*p* ≤ 0.05) pasting viscosities compared to their native and MI-treated counterparts. However, the PT of dual-modified LRS was increased significantly (*p* ≤ 0.05). The degradation of the starch chain during dual treatment resulted in a significant drop in starch swelling power, resulting in a lower PV of dual-modified LRS. Aaliya et al. [15] also stated comparable results with dual-modified talipot stem starch. The lower PV further resulting in lower BDV indicated that dual-modified LRS samples also possess higher resistance to shear thinning upon cooking, making them an ideal component to prepare weaning foods [61]. The more significant decrease in the SBV and FV of dual-modified LRS can be attributed to the depolymerization of leached-out amylose and amylopectin, suggesting that dual treatments do not favor the reconstruction of starch chains. A reduction in the values of pasting properties can be advantageous for easy cooking and decreased retrogradation in starch-based foods [34].

### 3.10. Thermal Properties

The thermal properties of native, single-, and dual-modified LRS are shown in Table 3. Starch gelatinization is an irreversible heat-absorbing phase transition in which amylopectin double helixes dissociate from a semi-crystalline form to an amorphous structure. The gelatinization of the starch plays an essential role in food processing and is generally used to analyze its cooking qualities. It is suggested that starches with low gelatinization temperatures have better cooking characteristics [11]. The gelatinization temperatures (onset *T*_o_, peak *T*_p_, and conclusion *T*_c_) of native LRS were 28.9, 78.05, and 139.8 °C, respectively. In one earlier study, Sukhija et al. [8] reported *T*_o_, *T*_p_, and *T*_c_ of 68.30, 72.04, and 76.81 °C, respectively, for native LRS. The thermal characteristics of starch can be affected by many factors such as amylose–amylopectin ratio, chain distribution, double-helix order, and molecular arrangement of the crystalline region [48]. Compared with native LRS, *T*_o_ and *T*_c_ showed no significant (*p* ≤ 0.05) difference, but *T*_p_ increased significantly (*p* ≤ 0.05) after both single modifications and the highest value (86.7 °C) was observed for MI-treated LRS. This increase after MI treatment can be attributed to re-arrangement within the crystalline and amorphous regions in starch granules, which possibly strengthened the amylose–amylopectin and amylose–amylose interactions and resulted in the limited hydration and swelling of MI-treated starch [31]. Similar changes in the starch structure at the molecular level were also reported in other physical methods such as heat moisture treatments and annealing [62]. Some other studies also reported a similar shift to higher temperature after MI treatment [31]. After *γ*-irradiation, the significantly (*p* ≤ 0.05) increased *T*_p_ can be attributed to the formation of smaller molecules (e.g., monosaccharide and smaller-chain polysaccharide) due to degradation and is consistent with the increasing soluble content. The monosaccharides present in starches have been reported to elevate their gelatinization temperature [63]. Previously, cowpea starch irradiated up to 50 kGy also showed increased gelatinization temperature which could be due to a more disordered (decreased crystallinity) structure of starch granules [64].

Compared with native, *T*_o_ increased but *T*_c_ and *T*_p_ decreased for dual-modified (MI-IR and IR-MI) LRS. *T*_o_ and *T*_c_ are related to the melting temperatures of the weakest and strongest crystallization in starch, respectively [13]. After dual treatment, more severe breakage in starch granules and melting of some weak internal microcrystals leading to more stability can be attributed to the increase in *T*_o_. Additionally, different modifications leading to different compactness of starch granules can affect the gelatinization temperatures of modified starches [38].

The enthalpy of gelatinization (Δ*H_gel_*) measures the overall crystallinity of the amylopectin and indicates the loss of molecular order within the granule [11]. The Δ*H_gel_* of native LRS was 253.1 J/g (Table 3) and increased after single microwave or *γ*-irradiation treatment while it decreased after both dual (MI-IR and IR-MI) treatments. The increase in Δ*H_gel_* indicated that a single treatment disrupted the amorphous region of starch. On the other hand, a decrease in Δ*H*_gel_ can be attributed to the destruction of the double-helix structures [17], showing that dual treatments promoted the disruption of the more resistant crystalline area in LRS.

### 3.11. Dynamic Rheology

**Figure 6:**
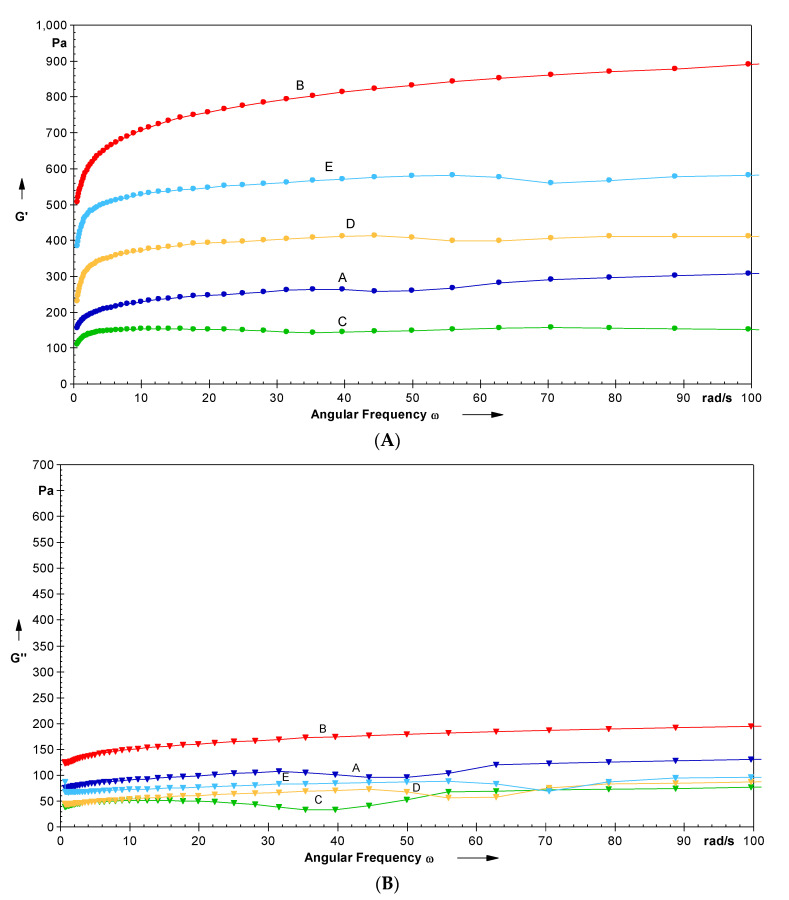
(**A**) Angular frequency dependence of G′ at 25 °C for native and modified lotus rhizome starches A—Native; B—MI; C—IR; D—MI-IR; E—IR-MI. (**B**) Angular frequency dependence of G″ at 25 °C for native and modified lotus rhizome starches A—Native; B—MI; C—IR; D—MI-IR; E—IR-MI.

The rheological behavior of the starch gel plays an important role in determining the processing ability, texture, and eating quality of foods prepared using different starches. The experimental data and frequency curves produced from frequency sweep tests are presented in Table 4 and Figure 6A,B, respectively. The elastic and viscous nature of the starch gel is indicated by rheological parameters G′ (dynamic storage modulus) and G″ (loss modulus), respectively. After every deformation cycle, G′ measures the energy recovered while G″ calculates the energy lost as viscous dissipation of the starch gel [65]. G′ and G″ values varied between 149.71 and 673.48 Pa and 49.22 and 143.29 Pa, respectively. The MI-treated LRS showed the highest while IR-treated LRS was found with the lowest G′ values (at 25 °C/6.28 rad/s). The rheological analysis showed G′ *>* G″ for starch gels of all LRS samples (native and modified), which revealed their elastic nature. The magnitude of G′ and G″ increased with increasing angular frequency; G″ showed a more significant increase than G′. No cross-over between G′ and G″ was observed, indicating the stability of these starch pastes over the applied frequency range (0.1–100 rad/s). After MI treatment, the value of G′ and G″ increased over the angular frequency range compared to native LRS. This increase can be due to microstructural and conformational changes in LRS due to gelatinization and cross-linking of the molecular chains leached out of the starch during MI processing [58]. A consistent observation for G′ and G″ was also reported in microwaved potato starch [58]. Upon IR treatment, LRS gel showed a decreased value of G′ and G″ compared to native LRS, which indicated a fragile and soft starch gel. The free-radicals-induced starch depolymerization decreased the gelling capacity and retrogradation ability of starch pastes, resulting in a weak starch gel. A consistent result was also observed for IR-treated talipot stem starch [15].

Both dual modifications showed a significant (*p* ≤ 0.05) increase in G′ but a significant (*p* ≤ 0.05) decrease in G″ compared to native LRS. Additionally, both dual-modified LRS samples showed significantly (*p* ≤ 0.05) lower and higher values of G′ and G″ compared to microwaved and *γ*-irradiated LRS, respectively. The increase in the G′ value of dual-modified LRS might be attributed to the enhanced stability of the granular integrity caused by the bond strengthening in the swollen granules [66]. A tan δ < 1 reflects elastic behavior predominantly, whereas tan δ > 1 indicates the viscous behavior of starch gels. Native and modified LRS showed tan δ values ranging between 0.14 and 0.40, which revealed the prevalence of elasticity over viscosity.

## 4. Conclusions

The modification of LRS using thermal and non-thermal methods made a noticeable change in the characteristics of starch and starch gels. Nevertheless, the dual modification of LRS by combining microwave and *γ*-irradiation significantly changed the properties more than single modifications. A combined effect of microwave and *γ*-irradiation resulted in the development of fissures and dents on the surface of starch granules. Additionally, the treatments reduced the peak intensities of OH and CH_2_ groups in FTIR spectra. An increase in the light transmittance value suggested a low retrogradation rate for dual-modified starches, making them more suitable for food application at refrigeration and frozen temperatures. Dual-modified LRS also showed remarkably lower values for the gelatinization and pasting properties but improved stability to shear thinning during cooking and thermal processing.

In addition, some apparent changes in the starch characteristics after dual modification could be found owing to the sequence effect, such as amylose content decreasing more in MI-IR-treated LRS compared to IR-MI-treated LRS. The pasting properties of MI-IR starch were significantly lower than the IR-MI sample, whereas for transition temperatures, the trend was reversed. The rheological properties were also affected by modification sequence; the G′ and G″ of MI-IR LRS were lower than IR-MI starch, giving it a soft gel texture. From this study, it can be concluded that microwave and *γ*-irradiation can be effectively used for modulating different characteristics of native starches. Since the safe dose limit for *γ*-irradiation suitable for human consumption is 10 kGy, combining it with other treatments such as microwave enhances the effect and usage of *γ*-irradiation in starch modification synergistically. Therefore, it can be concluded that these modifications can be applied in combination at a lower dose level and can produce modified starches with significantly altered properties desirable for diverse food and non-food applications.

## Figures and Tables

**Table 1:** Amylose content, swelling power, and solubility of native and modified lotus rhizome starches.

Sample	Amylose Content (%)	Swelling Power (at 90 °C) (g/g)	Solubility (at 90 °C) (%)	WAC (g/g)	OAC (g/g)
Native	18.94 ± 0.19 ^a^	23.82 ± 0.16 ^a^	9.63 ± 0.13 ^e^	1.62 ± 0.1 ^e^	1.30 ± 0.03 ^e^
MI	14.68 ± 0.21 ^d^	19.50 ± 0.13 ^b^	12.51 ± 0.09 ^d^	1.78 ± 0.03 ^d^	1.41 ± 0.02 ^d^
IR	16.55 ± 0.24 ^c^	4.13 ± 0.14 ^d^	86.9 ± 0.20 ^a^	1.88 ± 0.02 ^c^	1.53 ± 0.04 ^c^
MI-IR	17.58 ± 0.23 ^b^	10 ± 0.15 ^c^	79.5 ± 0.15 ^b^	1.95 ± 0.01 ^b^	1.89 ± 0.01 ^a^
IR-MI	18.86 ± 0.24 ^a^	9.71 ± 0.14 ^c^	78.5 ± 0.17 ^c^	2.00 ± 0.01 ^a^	1.78 ± 0.01 ^b^

MI: Microwaved, IR: Irradiated, MI-IR: Microwaved–irradiated, IR-MI: Irradiated–microwaved; WAC: Water absorption capacity; OAC: Oil absorption capacity. a–e: Means ± SD within the column with different lowercase superscripts are significantly different (*p* < 0.05).

**Table 2:** Pasting properties of native and modified lotus rhizome starches.

Sample	PV (mPa.s)	TV (mPa.s)	BDV (mPa.s)	FV (mPa.s)	SBV (mPa.s)	PT (°C)
Native	4486 ± 35 ^a^	1834 ± 25 ^b^	2652 ± 23 ^a^	2791 ± 21 ^b^	957 ± 15 ^a^	74.3 ± 0.1 ^c^
MI	3880 ± 33 ^b^	2384 ± 29 ^a^	1496 ± 26 ^b^	3095 ± 32 ^a^	711 ± 16 ^b^	76.9 ± 0.2 ^a^
IR	649 ± 13 ^e^	36 ± 2 ^e^	613 ± 14 ^e^	63 ± 11 ^e^	26 ± 3 ^d^	71.2 ± 0.1 ^d^
MI-IR	706 ± 18 ^d^	69 ± 11 ^d^	636 ± 17 ^d^	99 ± 12 ^d^	29 ± 2 ^d^	74.9 ± 0.2 ^c^
IR-MI	847 ± 15 ^c^	105 ± 10 ^c^	742 ± 15 ^c^	156 ± 12 ^c^	51 ± 4 ^c^	75.1 ± 0.3 ^b^

PV: Peak viscosity, TV: Trough viscosity, BDV: Breakdown viscosity, FV: Final viscosity, SBV: Set back viscosity, PT: Pasting temperature. a–e: Means ± SD within the column with different lowercase superscripts are significantly different (*p* < 0.05).

**Table 3:** Thermal properties of native and modified lotus rhizome starches.

Sample	*T*_o_ (°C)	*T*_p_ (°C)	*T*_c_ (°C)	Δ*H*_gel_ (J/g)
Native	28.9 ± 0.2 ^d^	78.1 ± 0.5 ^c^	139.8 ± 0.3 ^a^	253.1 ± 0.2 ^c^
MI	29.2 ± 0.3 ^cd^	86.7 ± 0.4 ^a^	140.5 ± 0.5 ^a^	323.7 ± 0.3 ^a^
IR	29.6 ± 0.5 ^bc^	82.6 ± 0.3 ^b^	140.2 ± 0.2 ^a^	287.6 ± 0.1 ^b^
MI-IR	31.3 ± 0.4 ^a^	77.3 ± 0.1 ^c^	130.4 ± 0.1 ^b^	199.7 ± 0.4 ^e^
IR-MI	30.3 ± 0.1 ^b^	72.9 ± 0.2 ^d^	125.1 ± 0.6 ^c^	228.0 ± 0.2 ^d^

*T*_o_: Onset temperature; *T*_p_: Peak temperature; *T*_c_: Conclusion temperature; Δ*H*_gel_: Enthalpy of gelatinization (dwb, based on starch weight). a–e: Means ± SD within the column with different lowercase superscripts are significantly different (*p* < 0.05).

**Table 4:** Dynamic rheological properties of native and modified lotus rhizome starches.

Sample	Storage Modulus G′ (Pa)	Loss Modulus G″ (Pa)	tanδ (G″/G′)
Native	216.00 ± 4 ^d^	86.64 ± 5 ^b^	0.40
MI	673.48 ± 8 ^a^	143.29 ± 6 ^a^	0.21
IR	149.71 ± 5 ^e^	49.22 ± 5 ^d^	0.33
MI-IR	357.41 ± 5 ^c^	50.16 ± 7 ^d^	0.14
IR-MI	511.96 ± 7 ^b^	70.09 ± 7 ^c^	0.14

a–e: Means ± SD within the column with different lowercase superscripts are significantly different (*p* < 0.05).

## Data Availability

The data presented in this study is available in this article.
